# lncRNA LOC100911717-targeting GAP43-mediated sympathetic remodeling after myocardial infarction in rats

**DOI:** 10.3389/fcvm.2022.1019435

**Published:** 2023-01-06

**Authors:** Pingjiang Li, Kang Wang, Jie Yin, Lei Qi, Hesheng Hu, Peijin Yang, Yugen Shi, Yan Li, Meng Feng, Hangji Lyu, Weili Ge, Xiaolu Li, Suhua Yan

**Affiliations:** ^1^Department of Cardiology, The First Affiliated Hospital of Shandong First Medical University and Shandong Provincial Qianfoshan Hospital, Shandong Medicine and Health Key Laboratory of Cardiac Electrophysiology and Arrhythmia, Jinan, China; ^2^Shandong First Medical University and Shandong Academy of Medical Sciences, Jinan, China; ^3^Department of Cardiology, Cheeloo College of Medicine, Shandong Qianfoshan Hospital, Shandong University, Jinan, China; ^4^Shandong Medicine and Health Key Laboratory of Cardiac Electrophysiology and Arrhythmia, Medical Research Center, The First Affiliated Hospital of Shandong First Medical University and Shandong Provincial Qianfoshan Hospital, Jinan, China; ^5^Department of Cardiology, Taizhou Hospital, Wenzhou Medical University, Taizhou, Zhejiang, China

**Keywords:** lncRNA LOC100911717, GAP43, macrophage, myocardial infarction, sympathetic neural remodeling

## Abstract

**Objective:**

Sympathetic remodeling after myocardial infarction (MI) is the primary cause of ventricular arrhythmias (VAs), leading to sudden cardiac death (SCD). M1-type macrophages are closely associated with inflammation and sympathetic remodeling after MI. Long noncoding RNAs (lncRNAs) are critical for the regulation of cardiovascular disease development. Therefore, this study aimed to identify the lncRNAs involved in MI and reveal a possible regulatory mechanism.

**Methods and results:**

M0- and M1-type macrophages were selected for sequencing and screened for differentially expressed lncRNAs. The data revealed that lncRNA LOC100911717 was upregulated in M1-type macrophages but not in M0-type macrophages. In addition, the lncRNA LOC100911717 was upregulated in heart tissues after MI. Furthermore, an RNA pull-down assay revealed that lncRNA LOC100911717 could interact with growth-associated protein 43 (GAP43). Essentially, immunofluorescence assays and programmed electrical stimulation demonstrated that GAP43 expression was suppressed and VA incidence was reduced after lncRNA LOC100911717 knockdown in rat hearts using an adeno-associated virus.

**Conclusions:**

We observed a novel relationship between lncRNA LOC100911717 and GAP43. After MI, lncRNA LOC100911717 was upregulated and GAP43 expression was enhanced, thus increasing the extent of sympathetic remodeling and the frequency of VA events. Consequently, silencing lncRNA LOC100911717 could reduce sympathetic remodeling and VAs.

## 1. Introduction

Ventricular arrhythmia (VA) is a common cause of sudden cardiac death after myocardial infarction (MI) (1). MI, a major cardiovascular disease (CVD), can lead to many complications, such as heart failure, cardiac fibrosis, sympathetic activation, and sympathetic remodeling. Studies revealed that gene mutations, epigenetic modifications, including DNA methylation, and other regulatory mechanisms are involved in the occurrence and development of post-MI complications (2–7). A cardiac sympathetic imbalance caused by post-MI sympathetic remodeling is a primary cause of VAs (8–10). Therefore, it is essential to reduce sympathetic remodeling after MI to improve patient prognosis.

A growing body of evidence suggests that sympathetic remodeling after MI is closely associated with inflammation (11–13) and mainly occurs at the periphery of the infarction, where inflammatory cells gather and regulate nerve remodeling by secreting nerve growth factor (NGF). Previous studies revealed that M1 macrophages can promote inflammation and play an important role in regulating sympathetic remodeling (14–17). According to a previous report, inhibition of M1 macrophage function could decrease NGF synthesis and improve sympathetic remodeling (18). This evidence reveals that M1 macrophages play a crucial role in sympathetic remodeling.

Long noncoding RNAs (lncRNAs) are longer than 200 nucleotides, cannot encode proteins, and play important roles in regulating apoptosis, proliferation, and migration (19–23). Furthermore, increasing evidence revealed that lncRNAs regulate macrophage polarization (24–26) and the occurrence and development of CVDs (27–30). Therefore, this study aimed to identify lncRNAs expressed in macrophages and involved in MI and determine possible regulatory mechanisms for lncRNAs in MI.

## 2. Materials and methods

### 2.1. RNA sequencing

RNA sequencing (RNA-seq) of lipopolysaccharide (LPS)- and interferon-gamma (IFN-γ)-stimulated RAW 264.7 and control cells was performed using the Illumina NovaSeq 6000 platform (Illumina, USA) to identify lncRNAs with different expression levels in M1 and M0 macrophages. Total RNA was extracted using the TRIzol reagent (Invitrogen, USA). RNA integrity was assessed using an Agilent Bioanalyzer 2100 (Agilent Technologies, Inc., USA). Strand-specific libraries were constructed using the VAHTS total RNA-seq (H/M/R) library prep kit (NR306-01; Vazyme, China) according to the manufacturer's instructions, and StringTie was used to count the fragments within each gene (31). Finally, a differential expression analysis of mRNAs and lncRNAs was performed using the edgeR package of R.

### 2.2. Gene ontology (GO) and kyoto encyclopedia of genes and genomes (KEGG) enrichment analyses of mRNA targets of lncRNAs

The potential functions of mRNA targets of lncRNAs were explored using the OmicShare tools (https://www.omicshare.com/tools). The mouse genome was a background reference to the GO and KEGG enrichment analyses of the mRNA targets of lncRNAs. We identified the GO terms, including the biological process (BP), molecular function (MF), and cellular component (CC) (*P* < 0.05), and the top 20 KEGG pathways (*P* < 0.05).

### 2.3. Homologous gene analyses

The UCSC LiftOver tool was used to analyze the lncRNA homologous regions in the rat genome, with the mouse genome as a background reference. The bedtools getfasta method was used to determine the lncRNA sequences in rats according to the homologous regions.

### 2.4. Cell culture and stimulation

RAW 264.7 cells were cultured in Dulbecco's modified Eagle's medium (Gibco, USA) supplemented with 10% fetal bovine serum (FBS; Gibco, USA) and NR8383 cells were cultured in Ham's F-12K medium (BasalMedia, China) supplemented with 20% FBS. Both cell lines were cultured at 37°C under a 5% CO_2_ atmosphere and stimulated as previously described (18) with LPS (10 μg/ml) and IFN-γ (20 ng/ml) for 12 h. Subsequently, the cells were collected for reverse transcription–polymerase chain reaction (RT-PCR) analysis.

### 2.5. Animals

Male Sprague–Dawley rats (7–8 weeks old; Vital River Company; Beijing, China) with an average weight of 270 g were used in this study. All the rats were housed in standard rat cages supplied with water and food *ad libitum* with a 12-h light/dark cycle at room temperature. All animal experiments followed the guidelines of the Animal Care and Use Committee of the First Affiliated Hospital of Shandong First Medical University (Approval number: No. 2020-S359).

### 2.6. MI model

All the rats were anesthetized using intraperitoneal injection of sodium pentobarbital (CSA: 76-74-4, 3%, 30 mg/kg) and were subjected to tracheal intubation. The hearts of the rats were exposed by cutting through the third and fourth ribs of the left thorax. The rat MI model was constructed by ligating the left anterior descending (LAD) branch of the coronary artery between the pulmonary artery cone and the left auricle, with a ligature 2–3 mm below the lower edge of the left auricle(32). The rat electrocardiogram (ECG) showed an ST-segment elevation, with the left anterior wall of the heart that turned pale, reduced motility (Supplementary Figure S1A), and a blotchy and pale appearance in the infarct areas (Supplementary Figure S1B), demonstrating the successful construction of the MI model. In addition, Masson staining was used to evaluate MI severity (Supplementary Figure S1C). The same operation was used in the sham group, but with only threading without ligation. After thorax closure, the rats were placed on a warming pad at 37°C and in a cage after regaining consciousness.

### 2.7. Adeno-associated virus infection in rats

All the rats were anesthetized using sodium pentobarbital as described previously. Then, tail vein adeno-associated virus (AAV) injections (1.5 × 10^13^ particles) were administered using an AAV with shlncRNA LOC100911717 or a control virus (AAV with shCtrl). The rats were divided into lncRNA LOC100911117 knockdown and control groups depending on the injected virus. The following three RNAi sequences targeting lncRNA LOC100911717 were used to ensure the gene silencing efficiency: GCTGCTGTCAGGGTGACATCT, GCTCCACGTCGGAATGCTAAG, and GCACCGCCCTCTGCATCCTTC, and the shCtrl sequence was TTCTCCGAACGTGTCACGT. The AAVs were constructed by Genomeditech (Shanghai, China) and they expressed the enhanced green fluorescent protein (eGFP) after 2 weeks. Thus, eGFP expression in the collected heart tissues indicated successful AAV transfection (Supplementary Figure S2A). Furthermore, RT-PCR was performed to verify the virus function (Supplementary Figure S2B).

### 2.8. Experimental design

#### 2.8.1. Protocol 1

Thirty rats that survived after the cardiac surgery were divided into three groups (*n* = 10 each) according to whether the LAD branch was ligated or not and depending on the time when the rats were sacrificed: (a) sham group; (b) MI 3d, 3 days after MI; and (c) MI 7d, seven days after MI. Total RNA was extracted from the heart tissue, and the lncRNA expression levels were detected using RT-PCR.

#### 2.8.2. Protocol 2

The rats were subjected to cardiac surgery 2 weeks after the AAV injection. The surviving rats were divided into four groups according to the type of surgery and the injected virus: (a) sham + shCtrl, sham-operated + AAV with shCtrl (*n* = 18); (b) sham + shlncRNA, sham-operated + AAV with shlncRNA LOC100911717 (*n* = 22); (c) MI + shCtrl, MI+ AAV with shCtrl (*n* = 20); and (d) MI + shlncRNA, MI + AAV with shlncRNA LOC100911717 (*n* = 18).

### 2.9. Tissue collection

The rats were sacrificed seven days postoperatively by injecting an overdose of 3% sodium pentobarbital, and 2 mm of myocardial tissue around the MI region of the left ventricle was collected. Heart tissues were either stored at −80 °C for further biochemical analysis or embedded in an optimal cutting temperature compound (OCT) and frozen for immunofluorescence.

### 2.10. RT-PCR

Total RNA was isolated using the TRIzol reagent and reverse transcription was performed using a PrimeScript™ RT kit (R323-01; Vazyme, China). Relative RNA expression levels were determined using a Bio-Rad iQ5 multicolor real-time PCR system (Bio-Rad Laboratories, USA) with SYBR Green (Q711; Vazyme, China). The 2^−Δ*ΔCt*^ method was used to calculate the relative targeted gene expression. Normalization was performed with GAPDH. The primer sequences are listed in Supplementary Table 1.

### 2.11. RNA pull-down assay

The RNA pull-down assay was performed using a GenSeq^®^ RNA Pull-Down Kit (GenSeq Inc., China), according to the manufacturer's instructions. A biotin-labeled “positive probe” or a “negative control probe,” whose sequence was reverse complementary to the positive probe, was mixed with streptavidin magnetic beads at room temperature for 30 min. Next, the probe-bound beads were incubated with the protein extracts of the samples at 4°C for 1 h and the bound proteins were recovered with an elution buffer. Finally, the retrieved proteins were separated *via* liquid chromatography using an Easy nLC 1000 system (ThermoFisher, USA) and analyzed using a Q-Exactive Orbitrap mass spectrometer (ThermoFisher, USA). The mass spectra were analyzed using the MaxQuant software (version 1.5.2.8).

### 2.12. Programmed electrical stimulation

Programmed electrical stimulation was performed in rats to determine their susceptibility to VAs. First, the rats were anesthetized with sodium pentobarbital, and then their hearts were exposed. Next, electrodes were placed on the left ventricular surface and the signals were recorded using an animal biofunctional experiment system. According to our previous study (33), the electrical stimulation procedure was performed with a cycle length of 120 ms with eight pacing beats (S0), followed by one to three additional stimulations (S1–S3). The stimulation was terminated when VAs were induced or when an effective refractory period occurred. The arrhythmia scores were calculated as previously described (32).

### 2.13. Heart rate variability (HRV) measurement

Rats were connected to an ECG machine, and the data were continually recorded using a PowerLab physiology system; 30-min ECG recordings were selected to analyze the HRV using the LabChart Pro software (AD Instruments). A low frequency (LF: 0.05–0.75 Hz) indicates parasympathetic and sympathetic tones, while a high frequency (HF: 0.75–2.5 Hz) indicates a parasympathetic tone. An LF/HF ratio increase indicated a cardiac sympathetic imbalance (34, 35).

### 2.14. Western blotting

Proteins were extracted from the heart tissue, and the concentrations were measured using a BCA protein colorimetric assay kit (E-BC-K165-M; Elabscience, China). The protein samples were separated on 12.5% SDS polyacrylamide gels and transferred onto polyvinylidene difluoride membranes (IPVH00010; Millipore, MA, USA). The membranes were blocked for 1 h in 5% non-fat milk diluted in TBS and then incubated with rabbit polyclonal anti-growth-associated protein 43 (GAP43) (GTX127937; GeneTex, 1:5,000) and rabbit monoclonal GAPDH (5174; Cell Signaling Technology, 1:1,000) primary antibodies overnight at 4°C and subsequently with a goat anti-rabbit IgG-HRP (abs20040; Absin, 1:5,000) secondary antibody for 1 h at room temperature. Protein bands were detected using an ECL chromogenic substrate (WBKLS0500, Millipore, USA) and analyzed using a chemiluminescence apparatus (Bio-Rad, USA).

### 2.15. Immunohistochemistry

Heart tissue was collected, embedded in paraffin, and cut into 5-μm sections. The tissue slices were incubated with a rabbit polyclonal anti-CD68 antibody (GB113109; Servicebio, China, 1:400) at 4°C overnight, followed by incubation with a goat anti-rabbit HRP-conjugated antibody (G1213; Servicebio, China, 1:200) for 1 h at room temperature. Finally, the slices were incubated with a DAB chromogenic kit (G1212; Servicebio, China) and counterstained with hematoxylin. The number of positive cells was observed under a microscope and calculated using the ImageJ software (version 1.8), and the mean was recorded for subsequent analysis.

### 2.16. Immunofluorescence staining

Heart tissue was cut into 7-μm-thick sections and fixed with acetone at 4°C for 10 min. Next, the sections were incubated with rabbit polyclonal anti-GAP43 (GTX127937; GeneTex, 1:200) and sheep polyclonal anti-tyrosine hydroxylase (TH) (AB1542; Millipore, 1:400) primary antibodies overnight at 4°C followed by incubation with Alexa 488-conjugated donkey anti-sheep (A-11015; Invitrogen, 1:400) and Alexa 594-conjugated donkey anti-rabbit (A-21207; Invitrogen, 1:400) secondary antibodies for 2 h at room temperature. Then, the cell nuclei were stained with DAPI (ab104139; Abcam, UK). Finally, the sections were blocked with an anti-fluorescence quencher. The areas of GAP43 and TH expressions were calculated using the ImageJ software (version 1.8).

### 2.17. Masson staining

Heart tissue was collected along the cross-section of the left ventricular infarction zone, embedded in paraffin, cut into 5-μm sections, and stained with a Masson's trichrome stain kit (G1346; Solarbio, Beijing, China) according to the manufacturer's instructions (Supplementary Figure S3). The infarct size was then calculated using the ImageJ software (version 1.8).

### 2.18. TUNEL staining

TUNEL staining was performed to assess the degree of myocardial apoptosis. Heart tissues were embedded in paraffin, cut into 5-μm sections, and stained using a DAB (SA-HRP) TUNEL cell apoptosis detection kit (G1507; Servicebio, China) according to the manufacturer's protocol. The proportion of the positive cells was calculated using the ImageJ software (version 1.8).

### 2.19. Hematoxylin-eosin (HE) staining

The histopathological examination of heart tissues was performed using HE staining. Heart samples were put in a 10% formaldehyde solution, dehydrated in an ethanol gradient, embedded in paraffin, and cut into 4-μm sections. After deparaffinization, the sections were stained with hematoxylin (G1005-1; Servicebio, China) and eosin (G1005-2; Servicebio, China) and then mounted and observed under a microscope (Olympus, Japan).

### 2.20. 2,3,5-Triphenyltetrazolium chloride (TTC) staining

Heart tissues were cut into 2 mm-thick sections and incubated with 2% TTC (G3005; Solarbio, Beijing, China) at 37°C for 30 min. After TTC staining, the surviving myocardia were red, and the infarct area was white. The infarct size was calculated using the ImageJ software (version 1.8).

### 2.21. Measurement of cardiac function by echocardiography

Measurements were performed on rats using an echocardiography machine (Fujifilm Vevo 3100, Japan). The left ventricular ejection fraction (EF%) and fractional shortening (FS%) were calculated using the M-model recording of the parasternal long-axis view.

### 2.22. Statistical analysis

Data were analyzed using the edgeR, heatmap, and ggplot2 packages in R and GraphPad Prism and expressed as mean±standard deviation. An unpaired Student's *t-*test compared differences between both groups. In addition, analysis of variance (ANOVA) compared more than two groups followed by Tukey's test. Statistical significance was set at a *P-*value of < 0.05.

## 3. Results

### 3.1. Identification of different lncRNA and mRNA expression profiles

**Figure 1:**
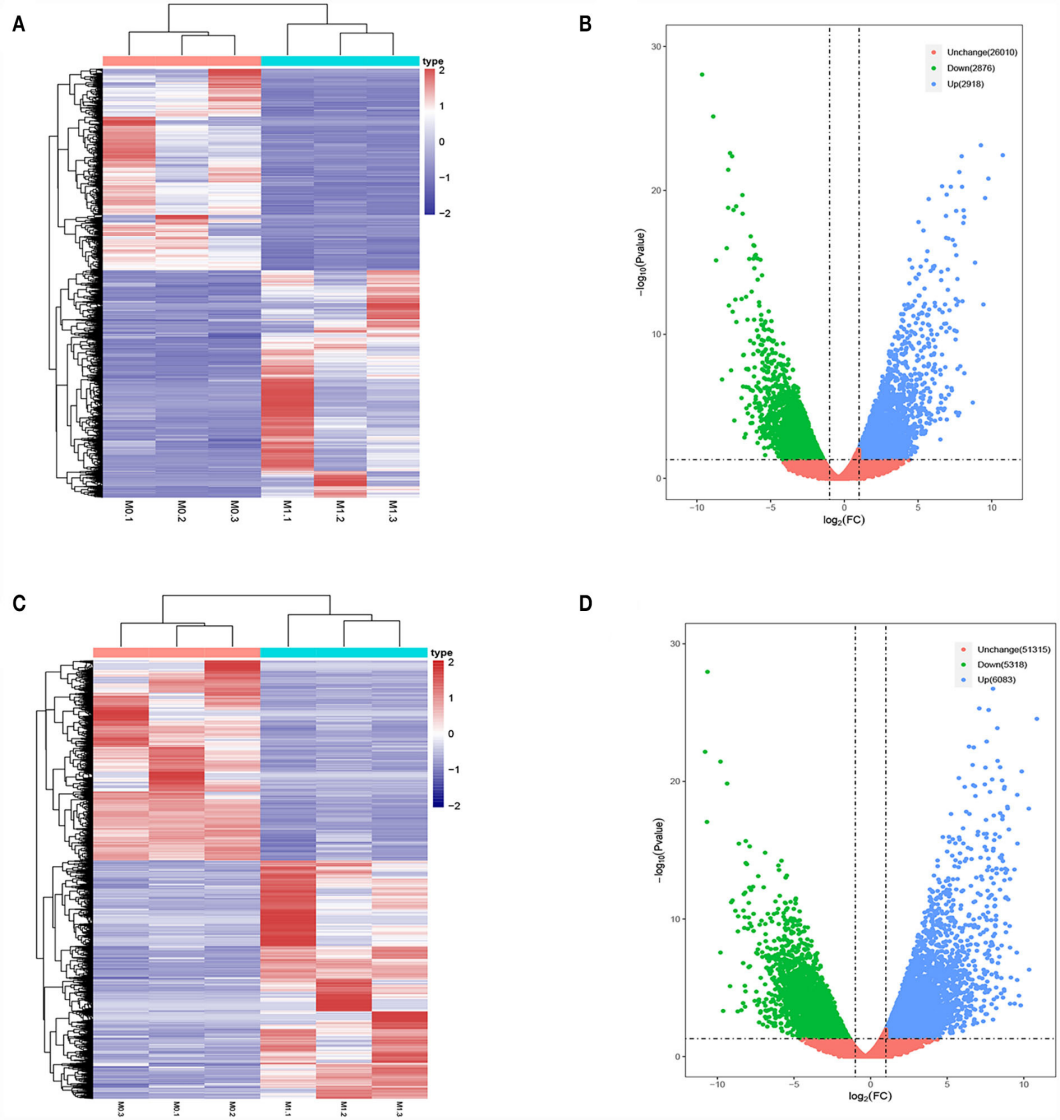
Differentially expressed lncRNAs and mRNAs in M0-type macrophages and M1-type macrophages. **(A)** lncRNAs Heat map. M0-type macrophages (M0-1, M0-2, and M0-3) and M1-type macrophages (M1-1, M1-2, and M1-3). **(B)** lncRNA Volcano plot. **(C)** mRNA Heat map. M0-type macrophages (M0-1, M0-2, and M0-3) and M1-type macrophages (M1-1, M1-2, and M1-3). **(D)** mRNA Volcano plot. Green or blue points represent upregulated or downregulated lncRNAs/mRNAs (log2 FC>1 and *P* < 0.05 and log2 FC <−1 and *P* < 0.05) and red indicates no significant difference.

Differences in the lncRNA and mRNA expression profiles were detected between M0- and M1-type macrophages using whole-transcriptome sequencing. According to the expression profiles, 5,794 lncRNAs (2,918 upregulated and 2,876 downregulated) and 11,401 mRNAs (6,083 upregulated and 5,318 downregulated) were differentially expressed (log2 FC>1 and *P* < 0.05 and log2 FC < −1 and *P* < 0.05). Figures 1A, B present the lncRNA heatmap and volcano plots and Figures 1C, D illustrate the mRNA heatmap and volcano plots.

### 3.2. GO and KEGG enrichment analyses of target mRNAs of upregulated lncRNAs

**Figure 2:**
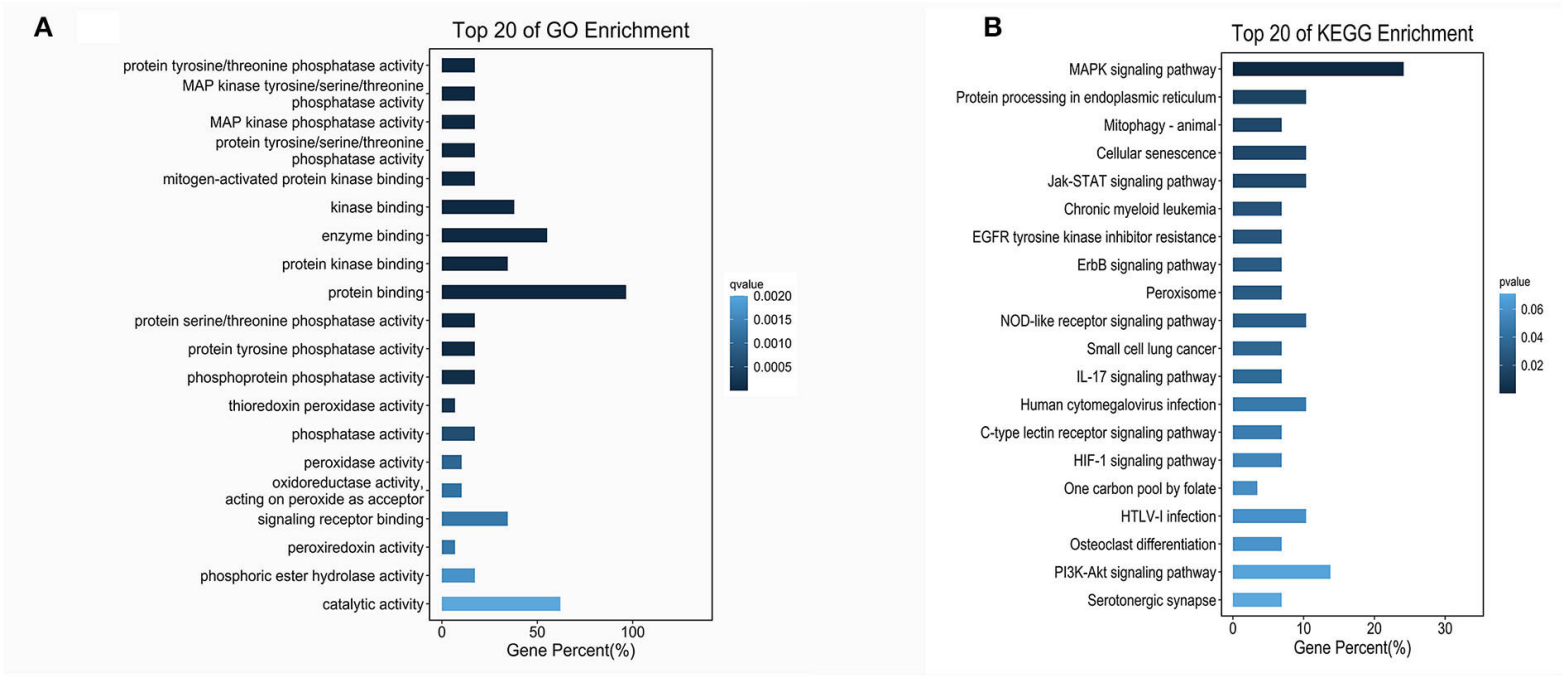
GO and KEGG enrichment of lncRNA target mRNAs. **(A)** The bar plot shows the top 20 GO enrichment results of lncRNA target mRNAs in MFs. **(B)** The bar plot shows the top 20 KEGG enrichment results of lncRNA target mRNAs.

According to the genomic locations of lncRNAs, their lengths and expression profiles were obtained. In total, 40 upregulated lncRNAs and their target mRNAs were screened (Supplementary Table 2). In addition, GO and KEGG enrichment analyses were performed to explore the potential functions of the target mRNAs of the upregulated lncRNAs. The GO enrichment analysis revealed that the target mRNAs of the lncRNAs were mainly enriched in the protein tyrosine/threonine phosphatase activity in the MF category (Figure 2A). The enrichment of KEGG pathways, including three inflammation-related pathways (the MAPK, JAK/STAT, and IL-17 signaling pathways), is illustrated in Figure 2B and the 10 lncRNAs are presented in Table 1.

**Table 1:** Different expression levels of lncRNAs in macrophages.

**lncRNA ID**	**AveExp**	**log2FC**	***Q*-value**
NONMMUT042032.2	4.26831713	7.143943624	<0.000001
NONMMUT147304.1	1.066735589	5.747978423	0.001879
ENSMUST00000181915	1.284953978	5.464138197	<0.000001
NONMMUT035084.2	3.500736986	4.463792591	<0.000001
ENSMUST00000181460	1.858593443	4.284292215	<0.000001
NONMMUT028804.2	1.851182601	3.999796624	0.000791
NONMMUT113494.1	3.060769578	3.779887996	0.000386
NONMMUT043538.2	3.51628241	3.446744258	0.000009
NONMMUT011901.2	2.492471252	3.367476373	0.000213
NONMMUT152633.1	2.655573976	2.998264175	0.000074

### 3.3. Upregulation of lncRNA Ptgs2os in M1-type macrophages and homologous gene analysis

**Figure 3:**
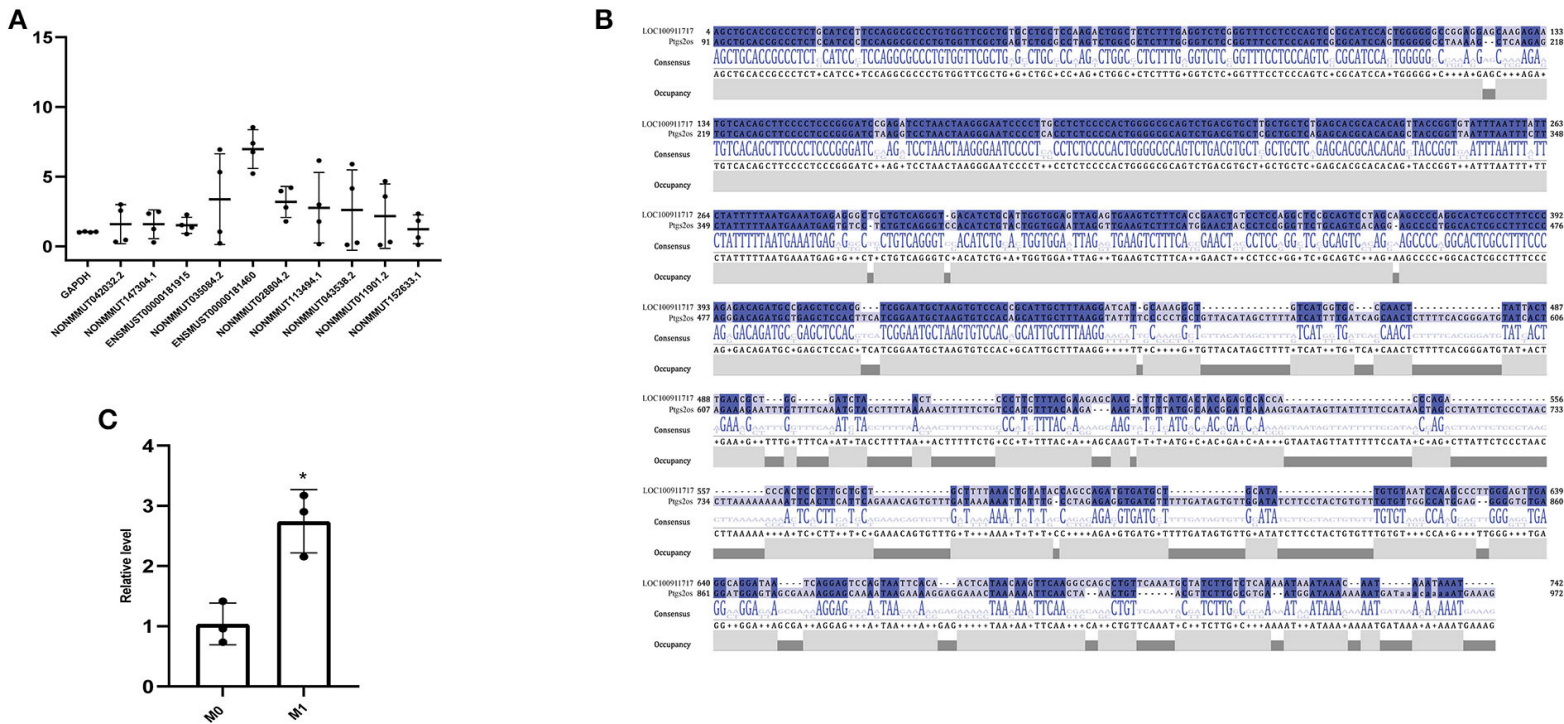
Comparison of lncRNA expression between the M0- and M1-type macrophages or between the sham group and the MI group and nucleotide sequence alignment. **(A)** Comparison of lncRNA expression between M0- and M1-type macrophages. **(B)** lncRNA LOC100911717 and lncRNA Ptgs2os nucleotide sequence alignment. **(C)** Comparison of lncRNA LOC100911717 expression between M0- and M1-type macrophages. *A *P-*value of <0.05 compared with M0-type macrophages.

Based on the expression profiles of lncRNAs from whole transcriptome sequencing and bioinformatics analysis, RT-PCR verified the expression levels of lncRNAs in M0- and M1-type macrophages. We observed that lncRNA Ptgs2os (Gene ID: ENSMUST00000181460) was highly expressed in M1-type macrophages (Figure 3A). According to a previous report, rat MI models mimic human MI better than mice MI models. They can easily reproduce the VA incidence (36). Therefore, upon homologous gene analysis from mice to rats, the lncRNA LOC100911717 is a homologous gene of lncRNA Ptgs2os in rats with 86.25% (389/451) alignment to the nucleotide sequence (Figure 3B). In addition, RT-PCR confirmed that lncRNA LOC100911717 was upregulated in rat M1-type macrophages (Figure 3C).

### 3.4. Increases in myocardial apoptosis, inflammation degree, and lncRNA LOC100911717 expression levels in rat hearts after MI

**Figure 4:**
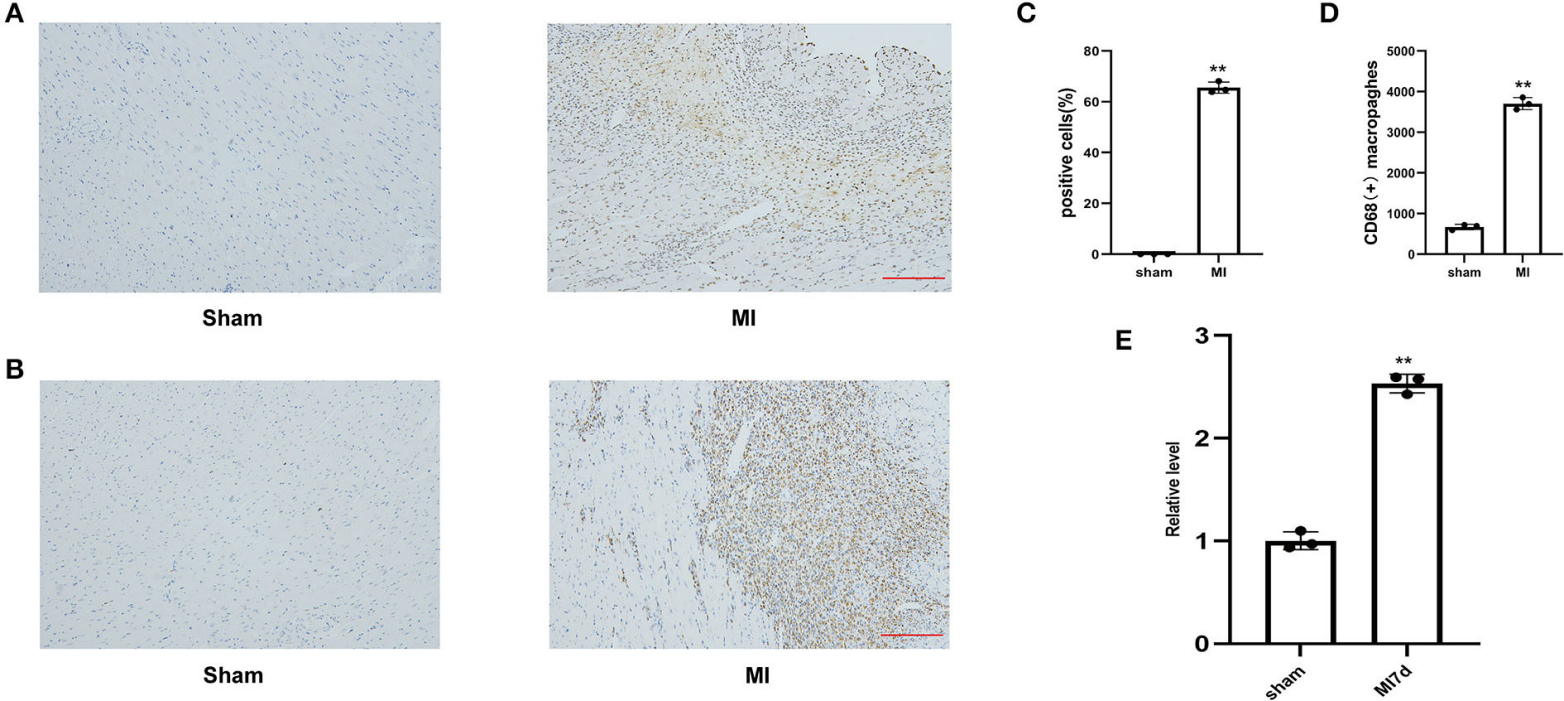
**(A)** Representative image of TUNEL staining of heart tissues, the amount of which reflects the degree of myocardial apoptosis. The apoptotic cells were stained brown and the neuron was stained blue (100HFP), bar = 100 μm. **(B)** Representative image of immunohistochemical staining for macrophage marker CD68 (brown) and nuclei (blue) (100HFP), bar = 100 μm. **(C)** The percentage of apoptotic cells at the infarcted border zone. **A *P-*value of <0.01 compared with the sham group. **(D)** Quantification of infiltrated macrophages at the infarcted border zone. **A *P-*value of <0.01 compared with the sham group. **(E)** Comparison of lncRNA LOC100911717 expression between the sham and MI7d groups. *n* = 3 per group. **A *P-*value of <0.01 compared with the sham group.

The percentage of TUNEL-positive cells and the number of CD68-positive macrophages in the MI and sham groups indicated the degrees of myocardial apoptosis and inflammation, respectively. The comparison of the MI and sham groups revealed that myocardial apoptosis and inflammation levels significantly increased in the infarcted border after MI (Figures 4A–D). The RT-PCR results revealed that the lncRNA LOC100911717 expression levels increased at 3 days and remained elevated at seven days post-MI compared with those in the sham group (Figure 4E).

### 3.5. Silencing of lncRNA LOC100911717 reduced GAP43 expression

**Figure 5:**
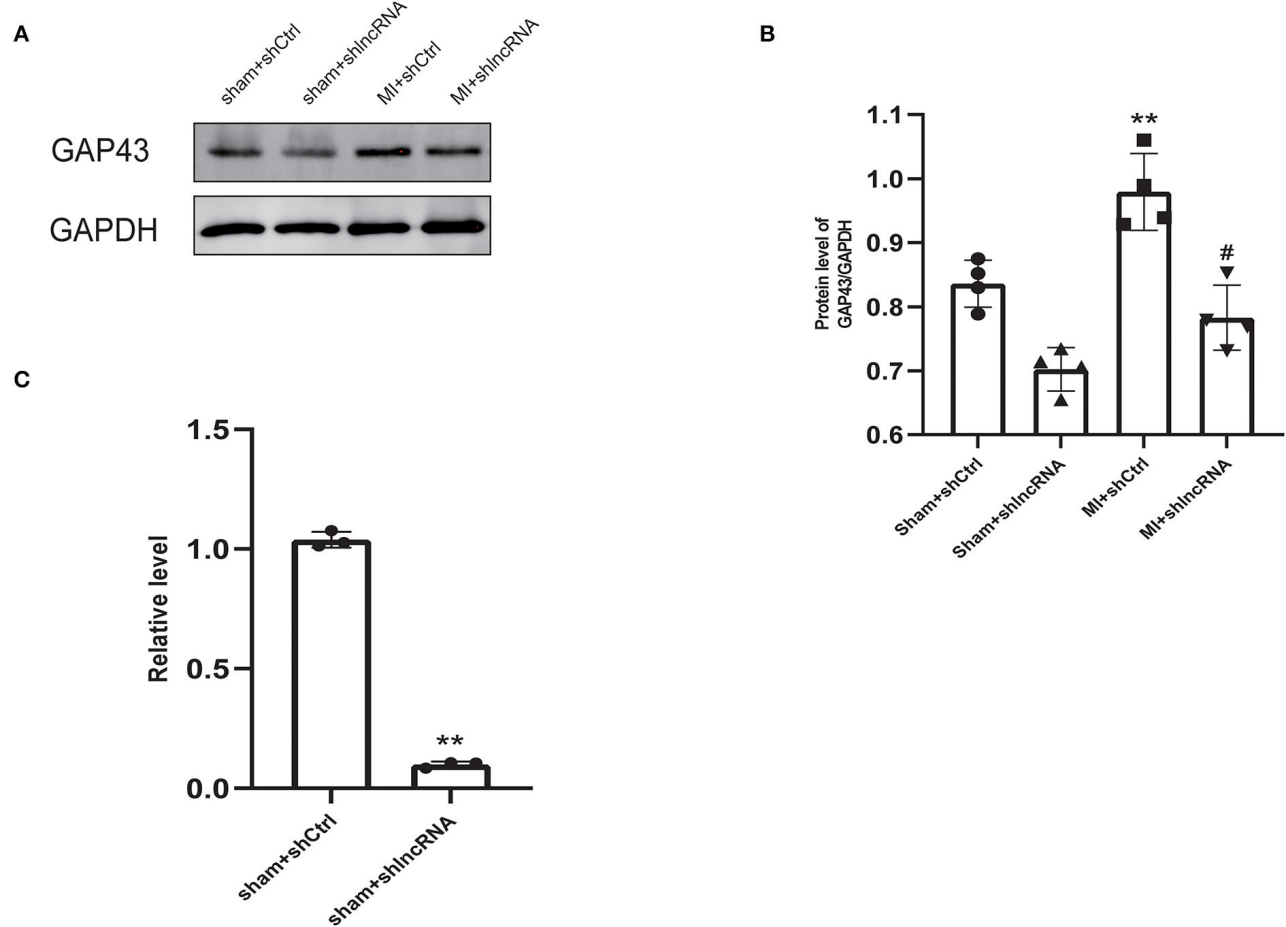
LncRNA LOC100911717 silencing inhibits GAP43 expression after MI. **(A)** Representative picture of the protein bands of MI myocardium from the four groups, sham+shCtrl, sham+shlncRNA, MI+ shCtrl, and MI+ shlncRNA; *n* = 4 per group. **(B)** Normalized GAP43 compared with GAPDH. **A *P-*value of <0.01 compared with the sham+shCtrl group and ^#^A *P-*value of <0.05 compared with the MI+ shCtrl group. **(C)** Comparison of GAP43 mRNA expression between the sham+shCtrl and sham+shlncRNA groups. *n* = 3 per group. **P < 0.01 compared with the sham group.

MI rat heart tissue was used for lncRNA LOC100911717 pull-down experiments. Mass spectrometry proteomics validated the pulled-down proteins. GAP43 was the first protein related to sympathetic remodeling among the top 10 proteins of the pull-down assay result (Supplementary Table 3). In addition, western blotting revealed that GAP43 was downregulated in the sham + shlncRNA group, while its level was significantly higher in the MI + shCtrl group than in the sham + shCtrl group and was reduced in the MI + shlncRNA group (Figures 5A, B). Meanwhile, silencing lncRNA LOC100911717 decreased *Gap43* mRNA expression in the sham + shlncRNA group compared with that in the sham + shCtrl group (Figure 5C).

### 3.6. Silencing of lncRNA LOC100911717 reduced sympathetic remodeling and decreased the susceptibility of rats to VAs post-MI

**Figure 6:**
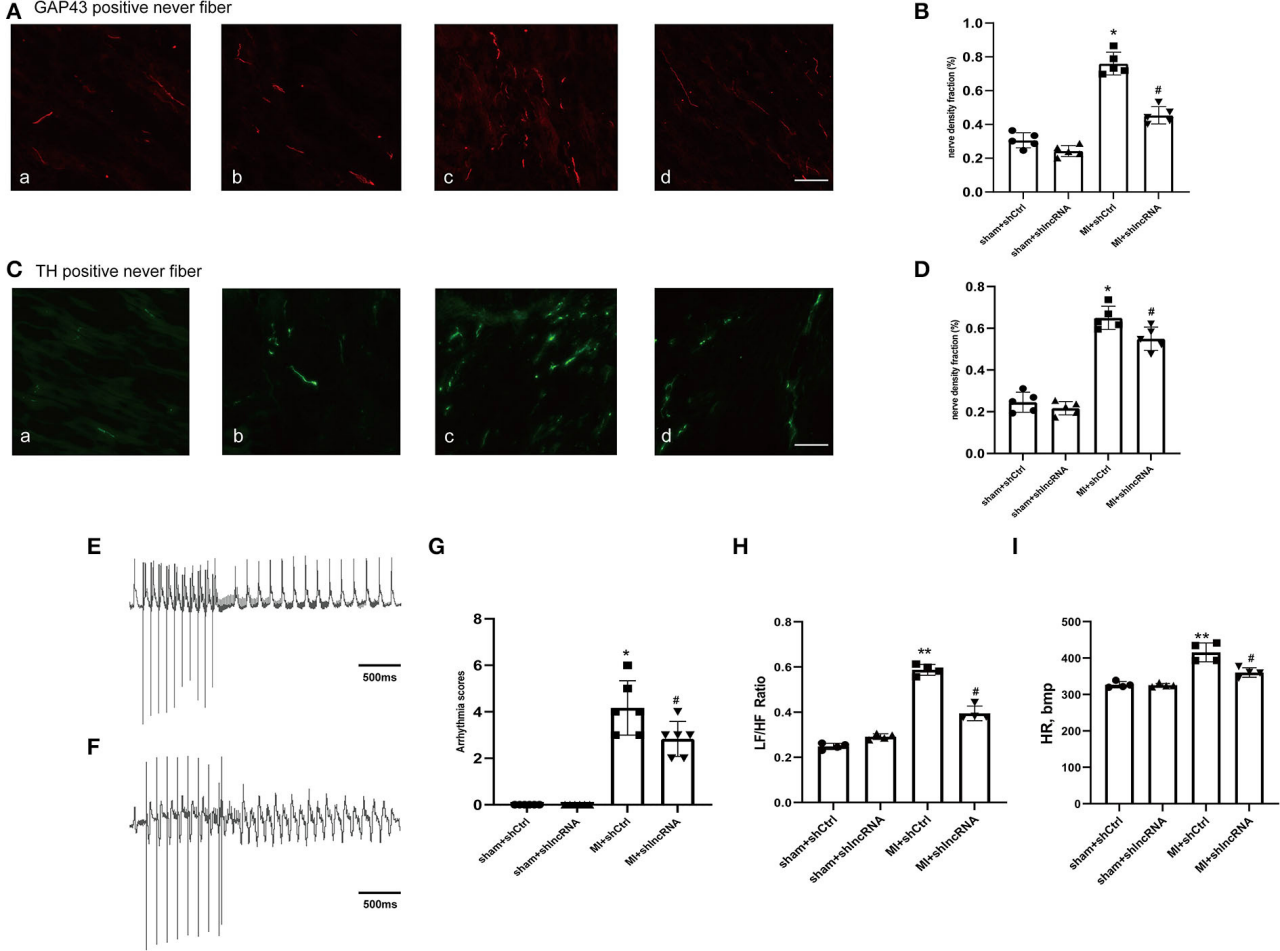
lncRNA LOC100911717 silencing reduces sympathetic remodeling after MI. **(A–C)** Immunofluorescence staining of GAP43 and TH in the MI border region (magnification 200 ×). (a) Sham+shCtrl, (b) sham+shlncRNA, (c) MI+ shCtrl, and (d) MI+ shlncRNA, bar = 50 μm. **(B–D)** Fraction (%) of nerve density area, *n* = 5 per group. **(E–F)** Normal electrocardiographic recordings and typical inducible VAs. **(G)** Arrhythmia scores from the four groups, sham+shCtrl, sham+shlncRNA, MI+ shCtrl, and MI+ shlncRNA; *n* = 6 per group. **(H–I**) Are statistical plots of the HRV analysis for each group of rats. *A *P-*value of <0.05 compared with the sham group, **A *P-*value of <0.01 compared with the sham group, ^#^A *P-*value of <0.05 compared with the MI+ shCtrl group.

In rat hearts, the sympathetic nerve density and morphology were assessed using immunofluorescence staining to further explore whether lncRNA LOC100911717 silencing participates in sympathetic sprouting and remodeling after MI. The density of GAP43 was significantly higher in the MI+shCtrl group than in the sham+shCtrl group, while it was reduced in the MI+shlncRNA (Figures 6A, B). Similarly, the density of TH was significantly reduced in the MI + shlncRNA group in which the lncRNA LOC100911717 was knocked down (Figures 6C, D). Furthermore, programmed electrical stimulation, which was performed to determine the susceptibility of rats to VAs, showed significantly higher arrhythmia scores in the MI + shCtrl group than in the sham groups. Meanwhile, the arrhythmia scores were significantly lower in the MI + shlncRNA group than in the MI + shCtrl group (Figures 6E–G). In addition, compared with the MI+shCtrl group, the MI+shlncRNA group had downregulated the LF/HF ratio (Figures 6H, I).

### 3.7. Silencing of lncRNA LOC100911717 reduced the infarcted heart area and improved cardiac function post-MI

**Figure 7:**
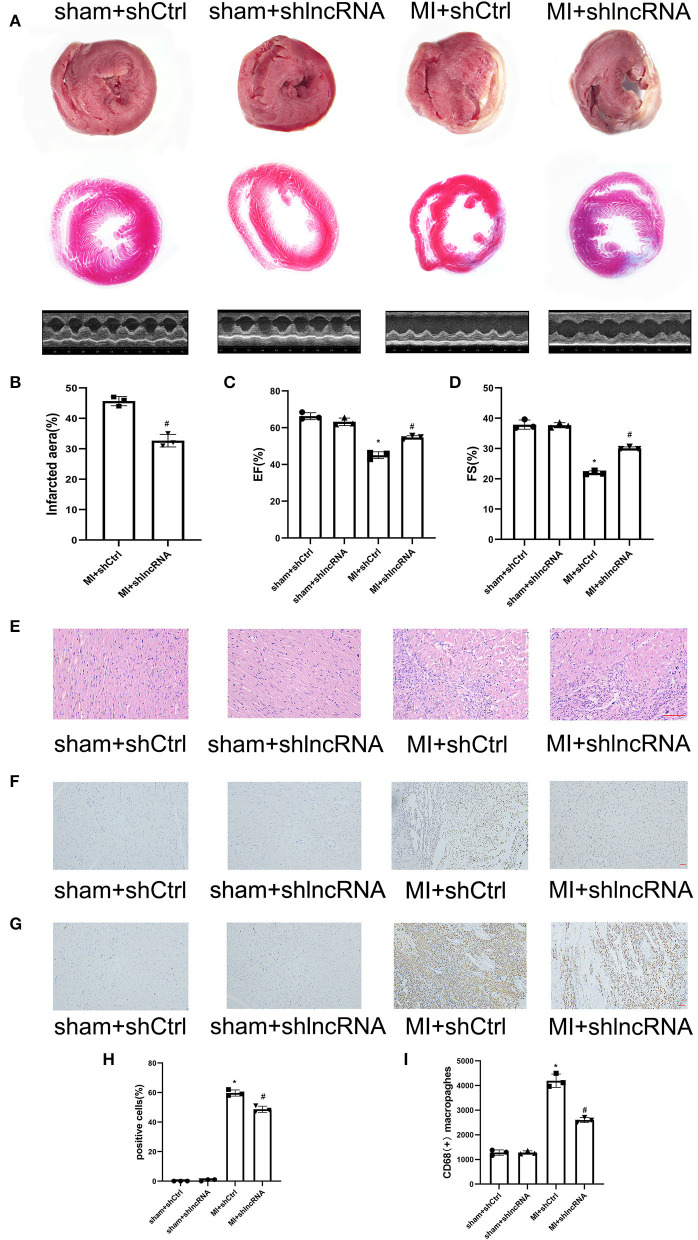
lncRNA LOC100911717 silencing improves cardiac function post-MI. **(A)** TTC staining, Masson staining, and parasternal long axis view of M-type echo of rat heart in the sham+shCtrl, sham+shlncRNA, MI+ shCtrl, and MI+ shlncRNA groups. **(B)** Comparisons of the infarcted area in the MI+ shCtrl and MI+ shlncRNA groups, *n* = 3 per group. **(C, D)** Comparisons of the left ventricular EF and left ventricular FS between the four groups, n = 3 per group. **(E)** The HE staining results showed that inhibition of the lncRNA LOC100911717 relieved heart tissue damage (100HFP), bar = 100 μm. **(F)** Representative image of TUNEL staining of heart tissue, the amount of which reflects the degree of myocardial apoptosis. The apoptotic cells were stained brown and the neuronal cells were stained blue (100HFP), bar = 50 μm. **(G)** Representative image of the immunohistochemical staining for macrophage marker CD68 (brown) and nuclei (blue) (100HFP), bar = 50 μm. **(H)** The percentage of apoptotic cells at the infarcted border zone between the four groups, *n* = 3 per group. **(I)** Quantification of infiltrated macrophages at the infarcted border zone between the four groups, *n* = 3 per group. *P < 0.05 compared with the sham group and ^#^*P* < 0.05 compared with the MI+ shCtrl group.

The MI size and cardiac function were also analyzed to determine the consequent effect of lncRNA LOC100911717 (Figures 7A–D). The infarct size in the MI + shlncRNA group was reduced compared to that in the MI + shCtrl group (Figures 7A, B). The echocardiography data revealed reduced EF% and FS% in the MI + shCtrl group vs. the sham + shCtrl group and the MI + shlncRNA group vs. the sham + shlncRNA group; however, the lncRNA LOC100911717 knockdown rescued EF% and FS% (Figures 7A, C, D). HE staining showed deformed nuclei and clear damage to the myocardium in the MI + shCtrl group. By contrast, these pathological changes were markedly improved in the MI + shlncRNA group (Figure 7E). Moreover, the percentage of apoptotic cells and the number of CD68-positive macrophages were higher in the MI + shCtrl group than in the sham + shCtrl group, and these increases were significantly attenuated by the lncRNA LOC100911717 knockdown (Figures 7F–I).

## 4. Discussion

Sympathetic remodeling is one of the most important causes of VAs after MI (37, 38). Therefore, it is important to explore the factors influencing sympathetic remodeling to reduce VA incidence and improve the prognosis of patients with MI. This study used bioinformatics analysis to screen for upregulated lncRNAs and explore their regulatory mechanisms in MI. Consequently, a new possible mechanism of lncRNA involvement in sympathetic remodeling after MI may have been found. The specific results of this study are as follows: (a) lncRNA LOC100911717 was upregulated in M1-type macrophages and in the infarct border zone after MI in rats; (b) the lncRNA LOC100911717 knockdown reduced GAP43 expression; (c) the lncRNA LOC100911717 knockdown decreased sympathetic remodeling and reduced the incidence of VAs; and (d) the lncRNA LOC100911717 knockdown reduced the infarcted heart area and improved post-MI cardiac function.

As a recognized neurogenesis marker, GAP43 is expressed during neuronal development and synaptogenesis. It plays a crucial role in axonal outgrowth and synaptic plasticity (39, 40). For instance, GAP43 expression can be elevated *via* the activation of the geniposidic acid PI3K/AKT pathway. It can improve nerve injury (41). In addition, a recent study demonstrated that GAP43 affects cancer development (42, 43). Carvedilol, a nonselective β-blocker, can suppress GAP43 expression and ameliorate sympathetic nerve sprouting and electrical remodeling after MI (44). The AAV-shlncRNA construct had an effect similar to that of carvedilol. As a novel diagnostic and therapeutic strategy, nanomaterial-based technology can be used for drug delivery (45). Therefore, the conjugation of lncRNA-silencing elements with nanoparticle drug carriers may lead to macrophage-targeting nanodrug development and contribute to new therapies. It is becoming increasingly clear that lncRNAs function through various mechanisms during the onset and progression of CVDs, such as MI and arrhythmia, cardiac remodeling, and heart failure (46–48). However, the potential molecular mechanism has not been cleared. This study utilized bioinformatics and molecular biology methods to explore and verify the biological functions of the lncRNA LOC100911717 and identified a new mechanism of lncRNAs as drivers of CVD development.

The data of this study indicated that the lncRNA LCO100911717 was upregulated after MI and could affect GAP43 expression. However, the lncRNA LOC100911717 knockdown suppressed GAP43 expression and sympathetic remodeling. These findings are significant because the lncRNA LOC100911717 knockdown decreased the susceptibility of rats to VAs, which is beneficial for MI prognosis. However, rescue experiments with GAP43 overexpression are needed to confirm the effect of lncRNA LOC100911717 on GAP43-mediated post-MI sympathetic remodeling. Furthermore, the pull-down mass spectrometry proteomics results showed that many proteins interacted with lncRNA LOC100911717. Hence, other proteins cannot be excluded as intermediate molecules participating in post-MI sympathetic remodeling. Therefore, future studies are needed to explore the detailed mechanism of GAP43 regulation and other biological functions of lncRNA LOC100911717.

### 4.1. Outlook

To the best of our knowledge, this is the first study to report that lncRNA LOC100911717 interacts with GAP43 and increases sympathetic remodeling after MI. In addition, this study identified a mechanism through which lncRNAs regulated post-MI sympathetic remodeling, thus improving the understanding of the pathogenesis of sympathetic remodeling. In this study, only interactions between lncRNA LOC100911717 and GAP43 were verified; consequently, the participation of other molecules should be explored in future studies.

### 4.2. Limitations

This study's data were obtained in animal models, and there may be human differences. Hence, these data should be cautiously extrapolated to humans. In addition, a tail vein injection of AAV-shlncRNA caused lncRNA LOC100911717 silencing in many types of rat tissues, not only in the heart, which may have led to a bias in the results.

## 5. Conclusion

**Figure 8:**
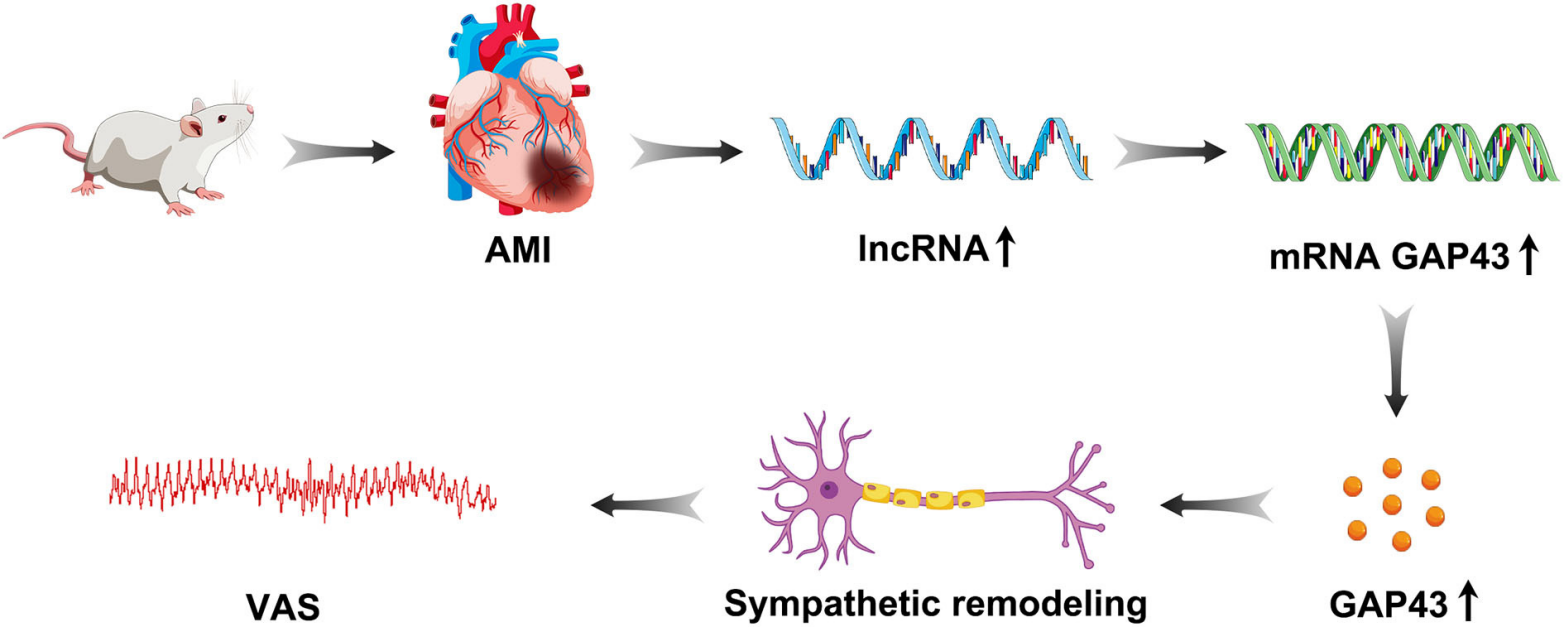
Graphical representation of lncRNA LOC100911717 functions in sympathetic remodeling after MI.

This study demonstrated that lncRNA LOC100911717 was upregulated and enhanced GAP43 levels post-MI expression, thereby increasing post-MI sympathetic remodeling and the incidence of VA events (Figure 8). Conversely, silencing the lncRNA LOC100911717 reduced sympathetic remodeling and the incidence of VAs. These results pave the way for further studies investigating lncRNAs that drive sympathetic remodeling after MI.

## Data availability statement

Publicly available datasets were analyzed in this study. This data can be found here: https://www.ncbi.nlm.nih.gov/bioproject/PRJNA866699.

## Ethics statement

The animal study was reviewed and approved by Animal Care and Use Committee of the First Affiliated Hospital of Shandong First Medical University.

## Author contributions

Conceptualization: SY and PL. Methodology: KW. Software: JY. Validation: KW, LQ, and HH. Formal analysis and visualization: PY. Investigation: YL. Resources: MF. Data curation: HL and JY. Writing—original draft preparation: WG. Writing—review and editing: PL and XL. Supervision: PL and KW. Project administration: SY. Funding acquisition: SY and YS. All authors have made important contributions to the revision and approval of the final manuscript.

## Supplementary material

Supplementary Figure S1 **(A)** ST-segment elevation ECG of rat after MI. **(B)** Representative image of rat hearts after MI. **(C)** Masson staining of MI heart tissue.

Supplementary Figure S2 **(A)** Enhanced green fluorescent protein (eGFP) expressed in heart tissue. **(B)** lncRNA 100911717 expression level of the six groups; the sham, sham+shCtrl, sham+shlncRNA, MI7d, MI+shCtrl, and MI+shlncRNA groups, *n* = 3 per group.

Supplementary Figure S3 Masson staining of MI heart tissue.

Supplementary Table 1 Primers used for RT-PCR.

Supplementary Table 2 40 up-regulated lncRNAs target mRNAs.

Supplementary Table 3 Top 10 proteins of RNA pull-down results.
