# Supplementary figures and images for: lncRNA LOC100911717-targeting GAP43-mediated sympathetic remodeling after myocardial infarction in rats
Source: Front Cardiovasc Med. 2023 Jan 6;9:1019435. doi: 10.3389/fcvm.2022.1019435 (PMC9859628; doi:10.3389/fcvm.2022.1019435)

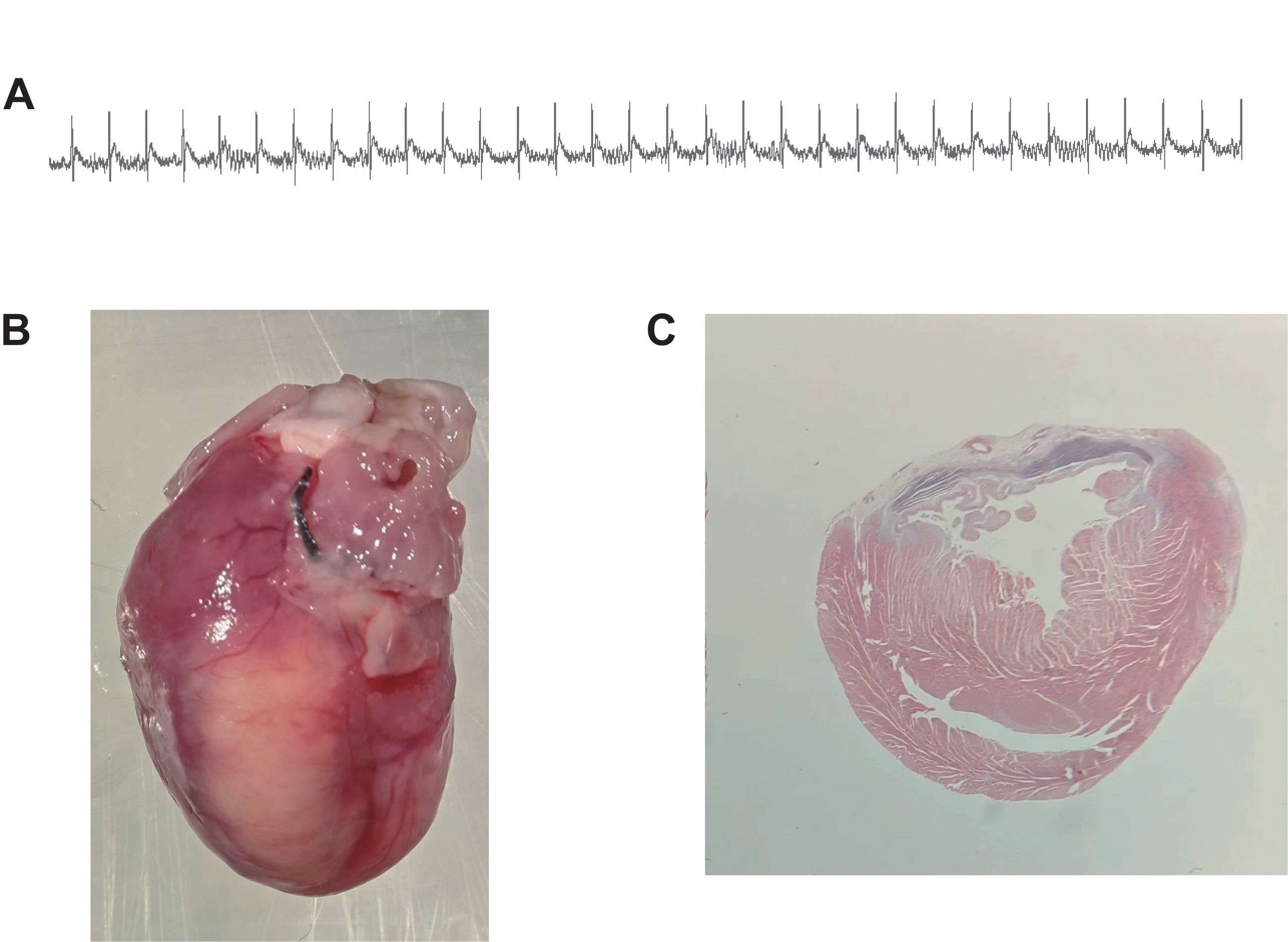

Supplement: Supplementary Figure S1 — (A) ST-segment elevation ECG of rat after MI. (B) Representative image of rat hearts after MI. (C) Masson staining of MI heart tissue. [file Image_1.TIF]

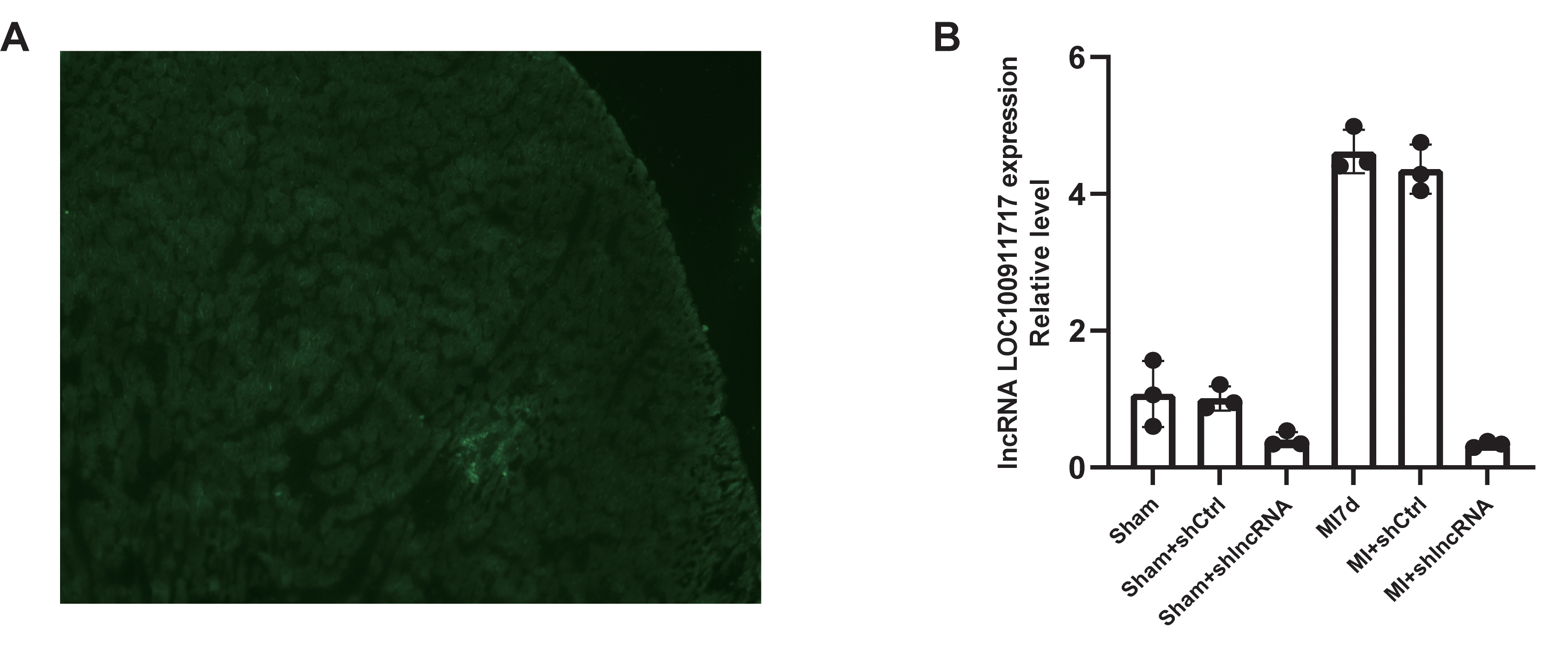

Supplement: Supplementary Figure S2 — (A) Enhanced green fluorescent protein (eGFP) expressed in heart tissue. (B) lncRNA 100911717 expression level of the six groups; the sham, sham+shCtrl, sham+shlncRNA, MI7d, MI+shCtrl, and MI+shlncRNA groups, n = 3 per group. [file Image_2.TIF]

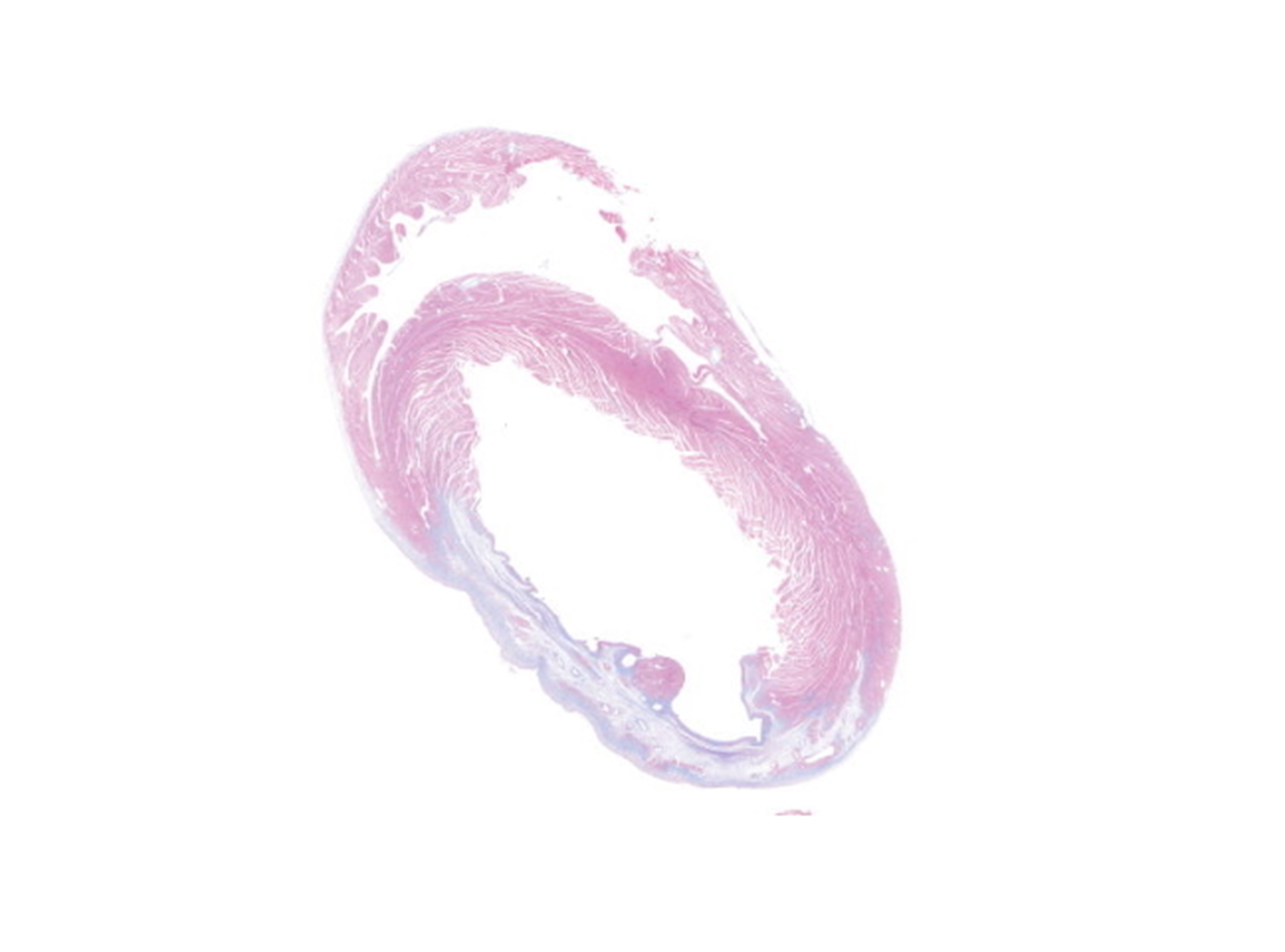

Supplement: Supplementary Figure S3 — Masson staining of MI heart tissue. [file Image_3.TIF]
