# Supplementary material for: lncRNA LOC100911717-targeting GAP43-mediated sympathetic remodeling after myocardial infarction in rats
Source: Front Cardiovasc Med. 2023 Jan 6;9:1019435. doi: 10.3389/fcvm.2022.1019435 (PMC9859628; doi:10.3389/fcvm.2022.1019435)
Supplement: Supplementary Table 1 — Primers used for RT-PCR. [file Data_Sheet_1.PDF]

Supplementary table 1 Primers used for RT-PCR

lncRNA ID	F	R
NONMMUT028804.2	5'-CTGAAGAGCCTTGTGTTCTAAGAC-3'	5'-CTGAAGCCTCATCGGATGGAT-3'
NONMMUT042032.2	5'-CTCTGGCAACTCTGTGAGACTGG-3'	5'-TTACATCGGCAGCAGAAG-3'
NONMMUT147304.1	5'-TTTGTGGCGGCTGTATTAAACTC-3'	5'-GGATGTTCTTTGACTCTCCTCTGA-3'
ENSMUST00000181915	5'-CATCTACCACACTTCCTGTTGACT-3'	5'-CCATCATCTCTGCCATCTCCAA-3'
ENSMUST00000181460	5'-GTGGTGGTGGATGGTGATGTTG-3'	5'-TGTGTATAGCTGGCTGTCCTGAA-3'
NONMMUT113494.1	5'-GTCAAGCAGAACTCGCACTGGA-3'	5'-TGCCTCGCTACTCAGGAACCT-3'
NONMMUT043538.2	5'-CCCTGGAACTCACTTTGTATGTCA-3'	5'-GCCATTGTGTCTCTGCTACCA-3'
NONMMUT035084.2	5'-AGGGCGTCTTAGAGCGAGAAA-3'	5'-GGGTGATGCGTGAGTGAGTCT-3'
NONMMUT152633.1	5'-CTCGAGAAACCTGACACTGT-3'	5'-GGCACGAAGAACGAGATTAAGTC-3'
NONMMUT011901.2	5'-GTGCTCAGATCAAGACTGCTTCT-3'	5'-CAGGTTCTTTCTTCTCTCGGGATG-3'
