# Supplementary material for: lncRNA LOC100911717-targeting GAP43-mediated sympathetic remodeling after myocardial infarction in rats
Source: Front Cardiovasc Med. 2023 Jan 6;9:1019435. doi: 10.3389/fcvm.2022.1019435 (PMC9859628; doi:10.3389/fcvm.2022.1019435)
Supplement: Supplementary Table 2 — 40 up-regulated lncRNAs target mRNAs. [file Data_Sheet_2.PDF]

Supplementary table 2 40 up-regulated lncRNAs target mRNAs

lncRNA ID	AveExpr	log2FC	Q.Value	lncRNA target mRNA
NONMMUT146777.1	1.050035883	2.7493291	0.023403	ENSMUSG00000026864
NONMMUT028804.2	1.851182601	3.999796624	0.000791	ENSMUSG00000024190
NONMMUT109157.1	1.016924141	2.889020217	0.070099	ENSMUSG00000027068
NONMMUT013107.2	1.814573973	2.967390763	0.000539	ENSMUSG00000025130
NONMMUT042748.2	1.129582973	2.553260204	0.013288	ENSMUSG00000025757
NONMMUT073066.2	4.182919631	3.359121123	0.000172	ENSMUSG00000025059
NONMMUT073068.2	2.966176217	2.843001841	0.000499	ENSMUSG00000025059
NONMMUT042032.2	4.26831713	7.143943624	0.000000	ENSMUSG00000040329
NONMMUT147304.1	1.066735589	5.747978423	0.001879	ENSMUSG00000040329
NONMMUT145793.1	59.19456136	3.817161168	0.000085	ENSMUSG00000024953
NONMMUT048868.2	18.8973454	2.572464789	0.000046	ENSMUSG00000028691
ENSMUST00000181915	1.284953978	5.464138197	0.000000	ENSMUSG00000032487
ENSMUST00000181460	1.858593443	4.284292215	0.000000	ENSMUSG00000032487
NONMMUT151781.1	1.302749947	2.107154238	0.016222	ENSMUSG00000004508
NONMMUT009591.2	7.955724055	3.522578542	0.000001	ENSMUSG00000015837
NONMMUT140454.1	1.527163021	2.297102282	0.015852	ENSMUSG00000020534
NONMMUT040551.2	4.468943063	2.05161596	0.000661	ENSMUSG00000007659
NONMMUT013862.2	1.324149007	2.551763012	0.024147	ENSMUSG00000020572
NONMMUT003877.2	1.270269643	2.860390713	0.000537	ENSMUSG00000073490
ENSMUST00000181915	1.284953978	5.464138197	0.000000	ENSMUSG00000032487
NONMMUT144855.1	8.236934147	2.264481659	0.000269	ENSMUSG00000061232
NONMMUT113494.1	3.060769578	3.779887996	0.000386	ENSMUSG00000062232
NONMMUT043538.2	3.51628241	3.446744258	0.000009	ENSMUSG00000062232
NONMMUT035084.2	3.500736986	4.463792591	0.000000	ENSMUSG00000034765
NONMMUT152633.1	2.655573976	2.998264175	0.000074	ENSMUSG00000031530
NONMMUT059175.2	1.902642473	2.917656719	0.000346	ENSMUSG00000030203
NONMMUT059174.2	1.236688064	2.737577371	0.000096	ENSMUSG00000030203
NONMMUT030758.2	1.066975336	2.46348982	0.001020	ENSMUSG00000024242
NONMMUT065205.2	55.47258502	2.153900791	0.000351	ENSMUSG00000031530
NONMMUT065206.2	3.131129135	2.1393294	0.030244	ENSMUSG00000031530
NONMMUT004166.2	1.099811672	2.125743772	0.008295	ENSMUSG00000039384
NONMMUT026915.2	1.428305958	2.273419101	0.017129	ENSMUSG00000022637
NONMMUT065069.2	7.068386298	2.300945304	0.014260	ENSMUSG00000031490
NONMMUT011901.2	2.492471252	3.367476373	0.000213	ENSMUSG00000038067

NONMMUT049178.2	1.658746143	5.180258488	0.000134	ENSMUSG00000028645
NONMMUT026005.2	4.226025044	2.659846882	0.000047	ENSMUSG00000022508
NONMMUT026003.2	4.128895348	2.246937023	0.002366	ENSMUSG00000022508
NONMMUT026004.2	4.244984188	2.056452489	0.005077	ENSMUSG00000022508
NONMMUT022925.2	2.002478841	2.363096241	0.000081	ENSMUSG00000022272
ENSMUST00000161524	2.219593674	2.12092822	0.011716	ENSMUSG00000022272
