# Supplementary material for: lncRNA LOC100911717-targeting GAP43-mediated sympathetic remodeling after myocardial infarction in rats
Source: Front Cardiovasc Med. 2023 Jan 6;9:1019435. doi: 10.3389/fcvm.2022.1019435 (PMC9859628; doi:10.3389/fcvm.2022.1019435)
Supplement: Supplementary Table 3 — Top 10 proteins of RNA pull-down results. [file Data_Sheet_3.PDF]

Supplementary table 3 Top 10 proteins of RNA pull-down results

Protein IDs	Gene names	Fold Change
M0RBX6	H3f3c	50.78267042
D3ZN79	Rpl35	32.33490994
P61016	Pln	29.61999587
P29418	Atp5e	25.9668558
D3ZEZ3	Dhrs2	25.37930083
Q642E2	Rpl28	24.05885906
F1LPQ8	Secisbp2	22.50382041
A0A0G2K808	-	21.98173615
P07936	Gap43	20.52270685
D3ZLY9	Hist1h2bl	19.8956568
